# Osteoporosis in Severe Asthmatic Patients: Data from the Severe Asthma Network in Italy (SANI) Registry

**DOI:** 10.3390/jcm14207387

**Published:** 2025-10-19

**Authors:** Manuela Latorre, Giulia Costanzo, Andrea Giovanni Ledda, Giada Sambugaro, Cristina Cardini, Isabella Sala, Chiara Oriecuia, Vincenzo Bagnardi, Francesco Blasi, Pierluigi Paggiaro, Giorgio Walter Canonica, Enrico Heffler, Gianenrico Senna, Davide Firinu, Ilaria Puxeddu, Laura Pini, Stefano Del Giacco, on behalf of the SANI Study Group

**Affiliations:** 1 Pulmonary Unit, UOC Pneumologia Nord, Toscana Nord Ovest, Nuovo Ospedale Apuano, 54100 Massa, Italy; manuela.latorre@yahoo.it; 2 Department of Public Health and Medical Science, University of Cagliari, 09124 Cagliari, Italy; andrea.giovanni.ledda@gmail.com (A.G.L.); giadasambugaro@gmail.com (G.S.); davidefirinu@yahoo.it (D.F.); delgiac@gmail.com (S.D.G.); 3 Fondazione per la Salute Respiratoria della Società Italiana di Pneumologia SIP-IRS, 20126 Milan, Italy; cristina.cardini@infomed-online.it; 4 Department of Statistics and Quantitative Methods, University of Milano-Bicocca, 20126 Milan, Italy; isabella.sala@unimib.it (I.S.); vincenzo.bagnardi@unimib.it (V.B.); 5 Department of Medicine and Surgery, University of Milano-Bicocca, 20126 Milan, Italy; 6 Department of Clinical and Experimental Sciences, University of Brescia, 25123 Brescia, Italy; chiara.oriecuia@unibs.it (C.O.); laura.pini@unibs.it (L.P.); 7 Department of Molecular and Translational Medicine, University of Brescia, 25123 Brescia, Italy; 8 Department of Pathophysiology and Transplantation, Università degli Studi di Milano, 20122 Milan, Italy; francesco.blasi@unimi.it; 9 Respiratory Unit and Cystic Fibrosis Center, Fondazione IRCCS Cà Granda Ospedale Maggiore Policlinico di Milano, 20122 Milan, Italy; 10 Department of Surgery, Medicine, Molecular Biology and Critical Care, University of Pisa, 56126 Pisa, Italy; pierluigi.paggiaro@unipi.it; 11 Personalized Medicine, Asthma and Allergy, Humanitas Clinical and Research Center, IRCCS, 20089 Rozzano, Italy; giorgio_walter.canonica@hunimed.eu (G.W.C.); enrico.heffler@hunimed.eu (E.H.); 12 Department of Biomedical Sciences, Humanitas University, Pieve Emanuele, 20072 Milan, Italy; 13 Department of Medicine, University of Verona, 37124 Verona, Italy; gianenrico.senna@univr.it; 14 Asthma Center and Allergy Unit, Verona University Hospital, 37134 Verona, Italy; 15 Immunoallergology Unit, Department of Clinical and Experimental Medicine, University of Pisa, 56126 Pisa, Italy; ilaria.puxeddu@unipi.it

**Keywords:** severe asthma, osteoporosis, Th2 inflammation, chronic rhinosinusitis with nasal polyps (CRSwNP), oral corticosteroids (OCS), bone health

## Abstract

**Background:** Severe asthma is associated with an increased risk of osteoporosis, largely due to chronic corticosteroid exposure and persistent systemic inflammation. Data from different international registries indicate a significant prevalence of osteoporosis among patients with severe asthma, with large variations attributed to differences in treatment strategies and optimization of care. **Aims and Methods**: This study aims to assess the prevalence of osteoporosis among patients with severe asthma enrolled in the Severe Asthma Network Italy (SANI) registry who are receiving treatment with monoclonal antibodies (mAbs) and/or long-acting muscarinic antagonists (LAMAs) and compare the characteristics of patients with and without osteoporosis to identify key risk factors contributing to osteoporosis. **Results**: A total of 1813 patients receiving Step 5 GINA (mAbs, LAMAs) treatment were included in the final analysis, of whom 282 (15.5%) had osteoporosis. Osteoporosis prevalence was significantly higher in women (20.3%) compared to men (8.0%). The prevalence also increased with age (*p* < 0.001) and with asthma duration (*p* = 0.008). Patients with osteoporosis exhibited poorer asthma control, lower lung function (FEV1 and FVC), a higher rate of exacerbations, and more frequent chronic oral corticosteroid (OCS) use compared to those without osteoporosis. Nasal polyposis was not significantly associated with osteoporosis in this cohort. **Conclusions**: Osteoporosis is highly prevalent in individuals with severe asthma, mainly due to chronic corticosteroid exposure and persistent inflammation, and is associated with asthma duration, sex, age, frequent exacerbations, cumulative exposure to OCS, and reduced lung function. Early recognition of osteoporosis risk is essential, and biologic therapies offer a promising strategy to reduce OCS dependence, mitigate adverse effects, and improve long-term outcomes.

## 1. Introduction

Osteoporosis is a condition characterized by weakened bones and an increased risk of fractures [1]. Although commonly linked to aging and hormonal changes, recent evidence highlights the significant role of inflammation in its development [2,3]. Indeed, chronic inflammatory diseases, including bronchial asthma, are associated with an increased risk of osteoporosis [4].

In individuals with asthma, the use of systemic and inhaled corticosteroids is a major risk factor for osteoporosis. Both high doses of inhaled corticosteroids (ICSs) and oral corticosteroids (OCSs) are associated with reduced bone mineral density (BMD) [5]. Several studies have also shown that patients with more severe or long-lasting asthma have a higher prevalence of osteoporosis and osteopenia, with long-term corticosteroid use significantly increasing this risk [6,7]. Although international asthma guidelines strongly recommend limiting the chronic use of OCSs, these medications are frequently used in patients with severe asthma to manage flare-ups and control persistent symptoms.

Biologic medications used for the treatment of severe asthma have been extensively proven to greatly enhance asthma management and can even lead most patients toward disease remission [8]. Similarly, other control therapies, such as long-acting muscarinic antagonists (LAMAs), have demonstrated effectiveness in improving asthma control in patients with more severe asthma [9]. However, these treatments are often introduced at a later stage of the disease, by which time prolonged and inappropriate use of corticosteroids may have already caused significant harm to bone health. Additionally, persistent inflammation in asthma elevates levels of pro-inflammatory cytokines, which disrupt bone remodeling and promote bone resorption [10]. However, the impact of severe asthma characteristics on osteoporosis remains insufficiently explored.

Data from both Italian and international registries indicate a significant prevalence of osteoporosis among patients with severe asthma, with reported rates ranging from 6.3% to 19.9%, depending on the registry. This wide variability across registries is largely attributed to differences in treatment strategies, with some patients being evaluated before receiving optimized care with inhaled medications and biologic therapies [11,12,13,14,15].

Our study aims to assess the prevalence of bone loss and osteoporosis among severe asthma patients enrolled in the Severe Asthma Network Italy (SANI) registry who are receiving Step 5 treatment per Global Initiative for Asthma (GINA) guidelines, which include high-dose ICSs usually combined with long-acting β2-agonists (LABAs), further optimized with the addition of LAMAs and/or monoclonal antibodies (mAbs). Additionally, we aim to describe and compare the characteristics of patients with and without osteoporosis in order to identify which asthma-related features are strongly associated with osteoporosis. By providing real-life evidence, our study aims to promote a more conscious and timely evaluation of biologic therapies, ultimately striving to reduce corticosteroid use and prevent disease progression.

## 2. Materials and Methods

### 2.1. Patient Recruitment

The study included all patients enrolled in the Severe Asthma Network Italy (SANI) registry who had a diagnosis of severe asthma as defined by GINA and/or ERS–ATS criteria. An essential inclusion criterion of the SANI protocol is the functional confirmation of asthma, as recommended by international guidelines, thereby reducing the likelihood of enrolling patients with other conditions that may resemble severe asthma. Patients with physician-diagnosed asthma–COPD overlap (ACO) were not specifically excluded, but only those fulfilling the specified criteria were included in the study.

From this cohort, only those who received GINA Step 5 therapy, including biologics approved for asthma treatment and/or LAMAs, were included. Patients were excluded if they lacked osteoporosis status information, had a missing baseline visit date (essential for evaluating treatment status in the SANI registry), or had been receiving treatment with biological drugs for more than six months at the time of the dual-energy X-ray absorptiometry (DXA) assessment.

Patients were stratified into two groups: those with a diagnosis of osteoporosis and those without a diagnosis of osteoporosis. Individuals who underwent DXA and demonstrated results consistent with osteoporosis, defined by the World Health Organization (WHO) criteria as a T-score ≤ −2.5, were classified as having osteoporosis.

For each participant, the following information was collected:

Demographic data: age, sex, height, weight, and body mass index (BMI).

Clinical features: age of asthma onset, duration of disease, allergic status, smoking history, and the presence of other comorbidities such as rhinitis, chronic rhinosinusitis without nasal polyps, nasal polyposis, and atopic dermatitis.

Asthma control was assessed according to the GINA guidelines, using standardized questionnaires such as the Asthma Control Test (ACT) and the Asthma Control Questionnaire (ACQ).

History of asthma exacerbations and frequency of unscheduled medical visits in the previous year.

Concomitant treatments: regular and on-demand treatments, including biological therapies and the chronic use of OCS.

Inflammatory markers: fractional exhaled nitric oxide (FeNO), blood eosinophil and neutrophil counts, and maximum blood eosinophil levels.

Pulmonary function tests: pre-bronchodilator forced expiratory volume in one second (FEV1) and forced vital capacity (FVC); Tiffeneau index (FEV1/FVC ratio).

Type 2 inflammation was defined by the presence of the following criteria: a blood eosinophil count greater than 150 cells/µL, a FeNO level above 25 ppb, and a documented history of allergic status.

### 2.2. Ethical Issues

This study complied with the Helsinki and Oviedo Declaration. The SANI registry was established in accordance with the third edition of the recommendation on registries for evaluating patient outcomes, published by the Effective Health Care Program of the Agency for Healthcare Research and Quality (https://effectivehealthcare.ahrq.gov/products/registries-guide-3rd-edition accessed on 6 October 2024). The protocol was designed following the principles and procedures of the Good Clinical Practice (ICH Harmonized Tripartite Guidelines for Good Clinical Practice 1996; Directive 91/507. EEC, The Rules Governing Medical Products in the European Community) and according to Italian law (D.L.vo n.211 24 June 2003 https://www.gazzettaufficiale.it/eli/gu/2003/08/09/184/so/130/sg/pdf accessed on 6 October 2024); D.L.n.200 6 November 2007 https://www.gazzettaufficiale.it/atto/serie_generale/caricaDettaglioAtto/originario?atto.dataPubblicazioneGazzetta=2007-11-09&atto.codiceRedazionale=007G0212 (accessed on 6 October 2024); MD, 21 December 2007 https://www.gazzettaufficiale.it/eli/id/2008/03/03/08A01360/sg (accessed on 6 October 2024). The study protocol was approved by the local Ethics Committee of Area Vasta NORD-OVEST Toscana, Italy (Protocol Number: 73714, 22 December 2016) and registered on ClinicalTrials.gov (ID NCT06625216; retrospectively registered on 3 October 2024).

### 2.3. Statistical Analysis

The data were presented stratified by the presence of osteoporosis. Continuous variables were expressed as means and standard deviations or medians and interquartile range (IQR), as appropriate, while dichotomous variables were represented as counts and percentages. Group comparisons were conducted using Student’s *t*-test for means, Wilcoxon test for medians, and Chi-squared test for proportions. In addition, we reported the prevalence of osteoporosis by sex and asthma onset time, as well as the prevalence of osteoporosis by age and asthma onset time, with corresponding statistical comparisons.

A multivariable logistic regression model was used to assess the association between chronic OCS use and poor asthma control with the prevalence of osteoporosis, adjusting for age, sex, and BMI. A two-tailed *p*-value < 0.05 was considered statistically significant. Statistical analyses were performed using SAS statistical software version 9.4.

## 3. Results

**Figure 1:**
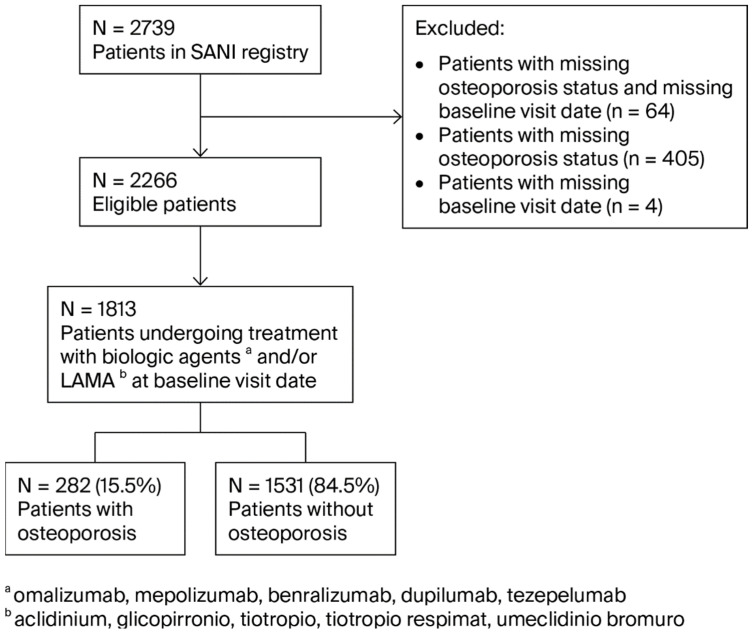
Flowchart of patient selection.

We initially screened 2739 patients included in the SANI registry, as shown in Figure 1. From this cohort, 473 patients were excluded due to missing osteoporosis status and/or missing baseline data. Out of the remaining 2266 eligible patients, we selected 1813 patients undergoing Step 5 GINA treatment.

This included high-dose ICSs, either as monotherapy or in combination with LABAs and/or LAMAs, such as aclidinium, glycopyrronium, tiotropium, tiotropium Respimat, or umeclidinium bromide.

Specifically, inhaled therapy was classified as dual therapy (ICSs + LABAs or ICSs + LAMAs) or triple therapy (ICSs + LABAs + LAMAs). The majority of patients received triple therapy, followed by dual therapy (ICSs + LAMAs). None received ICSs alone. Although all fulfilled the criteria for severe asthma, LABAs were discontinued for 54 patients for safety or tolerability reasons: 15 due to recurrence of atrial fibrillation, 20 due to persistent palpitations despite different molecules, and 8 for persistent tremors.

As these patients had severe asthma and their therapy had been optimized to the maximum extent possible, they were nonetheless included in the analysis (Table 1).

Additionally, biologic therapy was administered to a substantial proportion of patients, including mAbs such as omalizumab, mepolizumab, benralizumab, dupilumab, and tezepelumab (Table 1). The distribution of biologic therapies was similar between patients with and without osteoporosis, suggesting that treatment exposure was comparable across both groups.

Table 2 provides a summary of the key demographic and clinical characteristics of patients with and without osteoporosis.

Overall, 282 patients (15.5%) were diagnosed with osteoporosis, while 1531 (84.5%) did not have osteoporosis. The mean age was 63.2 ± 9.9 years in the osteoporosis (OP) group and 54.5 ± 13.4 years in the non-osteoporosis (nOP) group. Osteoporosis was significantly more prevalent among women (20.3%) compared to men (8.0%) (*p* < 0.001).

**Figure 2:**
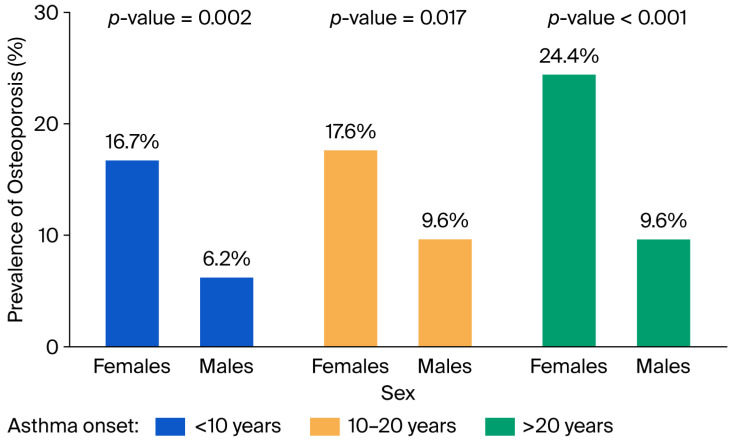
Prevalence of osteoporosis stratified by sex and time of asthma onset.

Women accounted for 80.1% of the osteoporosis group compared to 57.8% of the non-osteoporosis group. Age was also strongly associated with osteoporosis: the mean age of men with osteoporosis was 59.2 ± 11.6 years, compared to 55.0 ± 13.5 years in men without osteoporosis. In women, those with osteoporosis were older (63.5 ± 9.4 years) compared to those without (53.5 ± 13.3 years). The prevalence of osteoporosis increased with age (*p* < 0.001), with 4.3% of cases occurring in individuals under 50 years, 17.9% in those aged 50–70 years, and 27.9% in individuals over 70 years of age. Stratified by sex, similar trends were observed: in men, the prevalence was 4.1%, 9.5%, and 9.7%, respectively; in women, it was 4.4%, 23.2%, and 39.1% (Figure 2). The prevalence of osteoporosis increased with longer asthma duration, being 12.6% in patients with <10 years of disease, 14.4% in those with 10–20 years, and 19.0% in those with >20 years.

**Figure 3:**
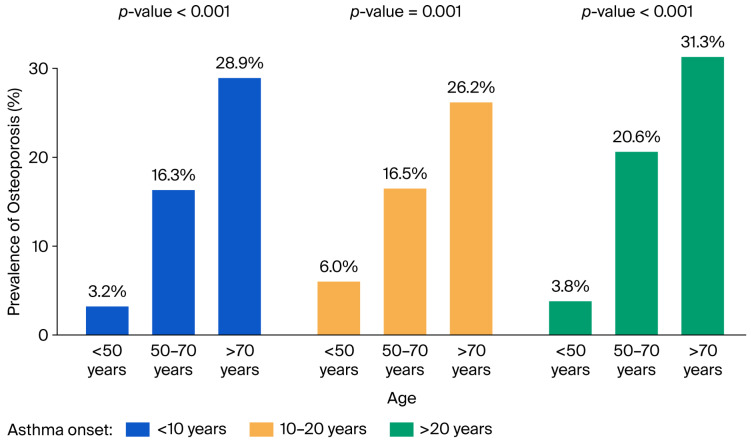
Prevalence of osteoporosis stratified by age and time of asthma onset.

The significant difference in osteoporosis prevalence between females and males, as well as among older patients, was confirmed regardless of asthma duration (Figure 2 and Figure 3).

Clinical features differed between groups. Patients with osteoporosis exhibited greater pulmonary function impairment compared to those without osteoporosis.

Specifically, patients in the OP group had significantly lower pre-bronchodilator FEV1, with an absolute mean of 1.7 ± 0.8 L, compared to 2.3 ± 2.1 L in the nOP group (*p* < 0.001). The percentage of predicted FEV1 was also significantly lower in the OP group (71.2% ± 25.1) compared to the nOP group (75.6% ± 21.5, *p* = 0.007). A similar trend was observed for FVC. The absolute mean FVC was 2.6 ± 1.0 L in the OP group, significantly lower than the 3.2 ± 1.0 L recorded in the nOP group (*p* < 0.001), and the percentage of predicted FVC was lower in the OP group (87.0% ± 24.9) compared to the nOP group (90.9% ± 20.3, *p* = 0.014).

Asthma control was poorer in the OP group, as indicated by significantly higher ACQ scores (2.5 ± 1.6 vs. 2.0 ± 1.6, *p* = 0.015). The median number of exacerbations in the previous 12 months was also higher among patients with osteoporosis, with a median of 2 (IQR 0–3) compared to 1 (IQR 0–3) in the nOP group (*p* = 0.002).

**Figure 4:**
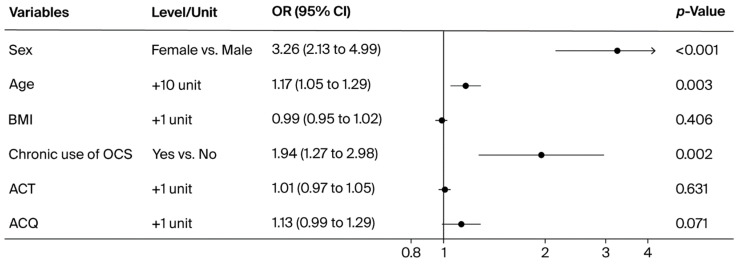
Multivariable logistic regression analysis to assess factors associated with osteoporosis, adjusted for age, sex, and BMI.

In the multivariable logistic regression model (Figure 4), chronic use of OCSs was independently associated with a higher prevalence of osteoporosis (OR 1.94, 95% CI 1.27–2.98, *p* = 0.002). Indicators of asthma control showed weaker associations: the ACT score was not significant (OR 1.01, 95% CI 0.97–1.05, *p* = 0.631), whereas the ACQ score showed a trend toward significance (OR 1.13, 95% CI 0.99–1.29, *p* = 0.071). As expected, demographic variables such as female sex (OR 3.26, 95% CI 2.13–4.99, *p* < 0.001) and older age (OR 1.17 per 10-year increase, 95% CI 1.05–1.29, *p* = 0.003) were also associated with osteoporosis, while BMI did not show a significant effect (*p* = 0.406).

Other Type 2 comorbidities, such as atopic dermatitis and allergic rhinitis, as well as chronic rhinosinusitis without nasal polyps, were not significantly associated with the prevalence of osteoporosis (Table 3). Similarly, no significant differences were observed between groups for other major comorbidities, including chronic rhinosinusitis with nasal polyps, obstructive sleep apnea syndrome (OSAS), gastroesophageal reflux disease, urticaria, psoriasis, aspirin/NSAID hypersensitivity, bronchiectasis, food allergy, eosinophilic granulomatosis with polyangiitis (EGPA), or alpha-1 antitrypsin deficiency. Endocrine and rheumatologic disorders were reported only in very few cases in both groups and therefore could not be meaningfully compared.

We did not find significant differences between the two groups in terms of blood eosinophils and neutrophils, FeNO, and Type 2 inflammation. Among the patients enrolled, a certain proportion were current or former smokers, with 4.5% current smokers and 26.6% former smokers, and a median smoking history of 10 (5–18) pack years. No significant differences in smoking status or pack years were observed between patients with and without osteoporosis.

## 4. Discussion

Our study highlights a strikingly higher prevalence of osteoporosis in patients with severe bronchial asthma undergoing treatment with mAbs and/or LAMAs compared with the general population. This finding is particularly relevant in a real-life setting, where patients already eligible for advanced therapies show a burden of osteoporosis well above national averages.

In Italy, osteoporosis is estimated to impact around 5 million individuals, with 80% being postmenopausal women. ISTAT (the Italian National Institute of Statistics) data from 2020 indicates that 8.1% of Italians reported having osteoporosis, comprising 13.5% of women and 2.3% of men. The condition’s prevalence rises with age, especially among women over 55 [16].

Several studies in the literature have examined the association between asthma and osteoporosis, consistently reporting a higher prevalence of osteoporosis in asthmatic patients, particularly in those with a longer disease history. A dose–response effect has also been observed, with greater exposure to OCSs or ICSs in the previous year being associated with an increased risk of osteoporosis and fragility fractures.

Two large epidemiological studies conducted using the UK Clinical Practice Research Datalink (CPRD) and related datasets have provided complementary insights into this relationship. In a matched cohort study, adults with an incident asthma code were identified and matched with up to four non-asthmatic controls by age and sex. Compared with the general population, patients with asthma had an 18% higher risk of osteoporosis and a 12% higher risk of fragility fractures. Importantly, a dose–response relationship was demonstrated for OCS and osteoporosis, and regular ICS use was associated with increased risk of both bone outcomes [17].

In parallel, two nested case–control studies using the CPRD and Hospital Episode Statistics (HES) database investigated the specific role of corticosteroid exposure. Patients with asthma and either osteoporosis or fragility fractures were compared with matched controls. A clear dose–response effect was observed: ≥9 OCS prescriptions per year increased the risk of osteoporosis 4.5-fold and fractures 2.2-fold, while ≥11 ICS prescriptions raised the risk 1.6- and 1.3-fold, respectively [18].

These findings highlight that OCS and ICS exposure are independent risk factors for impaired bone health in asthma, with effects modulated by age, cumulative dose, and fracture site.

Indeed, chronic use of OCSs can have a negative impact on bone health by decreasing calcium absorption, increasing calcium excretion, and directly affecting bone cells, thereby reducing bone formation and increasing bone resorption. This is especially concerning older adults or those with additional risk factors for osteoporosis [19].

While database studies provide strong epidemiological evidence, randomized controlled trials remain scarce, and real-life observational data on large populations are underpowered.

Recently, data from the International Severe Asthma Registry (ISAR) across 22 countries showed that severe asthmatic patients with osteoporosis were twice as likely to have long-term OCS exposure and more exacerbations compared with those without osteoporosis [12].

Nonetheless, no clear characterization of the severe asthmatic patient with osteoporosis—particularly those eligible for biologic therapies in real-world settings—has been proposed.

Our findings indicate that, in severe asthma cohorts, patients with osteoporosis—either eligible to start biologic therapy or who had only recently initiated it—are predominantly older women, highlighting the well-established link between age, sex, and bone health.

This is in line with other data from the literature, which consistently show that aging and hormonal changes—particularly estrogen decline during menopause—are major drivers of osteoporosis. Accelerated bone remodeling with age, compounded by estrogen deficiency, promotes increased resorption and reduced formation, ultimately leading to bone loss and microarchitectural deterioration [20].

Furthermore, the results of our study suggest a link between osteoporosis and asthma duration, asthma control, and corticosteroid exposure, highlighting a gap in current guidelines that do not consider osteoporosis risk when evaluating patients for biologic therapy. More intriguingly, our multivariable analysis revealed that chronic OCS use was independently associated with a higher prevalence of osteoporosis, even after adjustment for age, sex, and BMI, underscoring the clinical relevance of limiting long-term systemic corticosteroid exposure whenever possible.

Although current asthma guidelines recommend screening for osteoporosis, the risk of its future development—particularly in patients with factors such as longer disease duration, cumulative steroid exposure, sex, and age—is not explicitly addressed. These elements should instead support an earlier consideration of biologic therapy, aiming to achieve better disease control and reduce the overall corticosteroid burden, thereby lowering the long-term risk of osteoporosis.

Furthermore, of greater interest is the correlation between the presence of osteoporosis and asthma control. Patients with osteoporosis tend to have poorer control of current symptoms, as assessed by ACQ and ACT, and a higher “future risk” profile presenting greater bronchial obstruction, lower FEV1 values, and more frequent exacerbations.

These observations provide an interesting explanation for the correlation between osteoporosis and severe asthma of longer duration, particularly in cases with bronchial remodeling.

Indeed, asthma involves a complex interplay of inflammatory cells, cytokines, and mediators, resulting in airway hyperresponsiveness, mucus production, and structural changes in the airways. Inflammatory cells like eosinophils, T lymphocytes, mast cells, and neutrophils, along with cytokines and mediators such as interleukin-4 (IL-4), interleukin-5 (IL-5), interleukin-13 (IL-13), and tumor necrosis factor-alpha (TNF-α), play critical roles in perpetuating inflammation and recruiting additional inflammatory cells to the airways [21,22,23,24].

Chronic asthma inflammation can contribute to the development of osteoporosis by releasing pro-inflammatory cytokines into the systemic circulation [23]. These cytokines can interfere with bone remodeling by promoting bone resorption and inhibiting bone formation. In particular, TNF-α and interleukin-1 (IL-1) increase osteoclast activity, leading to greater bone breakdown [3,25]. Furthermore, persistent inflammation generates oxidative stress, which damages bone cells and impairs the function of the airways [26,27].

Both cytokines and pro-inflammatory factors involved in Type 1 and Type 2 inflammation can play similar roles in inducing the chronic inflammation that facilitates bone remodeling.

Therefore, it is unsurprising that there is no greater expression of Type 2 inflammation mediators among OP patients.

Several limitations of our study should be acknowledged. First, the inclusion of patients who had already initiated biological therapy may have obscured the expression of Type 2 biomarkers. However, given the similar distribution of biological therapies between the two groups, we believe that any bias in biomarker expression is also comparable when comparing patients with and without osteoporosis. A similar limitation may apply to the evaluation of asthma control over the previous year. However, this bias is minimized by excluding from the analysis patients who had been on biological therapy for more than six months. Second, cumulative OCS exposure could not be precisely calculated in a cohort of this size. Although data on the number of exacerbations, the type of corticosteroid, and the prescribed dose were collected, deriving a reliable estimate of cumulative exposure would be extremely challenging due to the heterogeneity in OCS formulations, treatment schedules, and dosages used during exacerbations. Moreover, the SANI registry records OCS use only for the year preceding enrollment, whereas lifetime exposure throughout the entire course of asthma would be more relevant for assessing its true impact on bone health. Despite this limitation, the observed association with asthma duration still provides robust evidence of the detrimental effects of prolonged exposure to OCSs and chronic high-dose ICSs on bone damage. Finally, our study did not include data on fractures or calcium/phosphate status, as these were not collected in the SANI registry. However, this limitation does not undermine the validity of our findings, which focused on the prevalence of osteoporosis and its association with patient characteristics in severe asthma.

Another important aspect to consider is that, in our cohort, a proportion of patients were former smokers, and a small percentage were current smokers; however, the overall smoking exposure remained limited, with a median history of 10 pack years. We agree that this is an important aspect to consider, as smoking and possible asthma–COPD overlap may influence disease characterization. Nevertheless, the inclusion criteria of the SANI registry required a confirmed diagnosis of severe asthma according to GINA/ERS/ATS guidelines, ensuring accurate case selection and minimizing the likelihood of enrolling subjects with predominant COPD or ACO asthma–COPD overlap.

One unexpected point highlighted by our study is that nasal polyposis does not correlate with the prevalence of osteoporosis in patients with severe asthma. Nasal polyposis is a chronic inflammatory condition characterized by persistent inflammation of the nasal mucosa. It is often present in patients with severe asthma and can cause significant symptoms such as nasal obstruction and loss of smell, leading patients to use inhaled and frequent oral corticosteroids [28].

While osteoporosis and nasal polyposis are distinct conditions, chronic inflammation is a common feature in both. Pro-inflammatory cytokines involved in nasal polyposis can also affect bone metabolism, potentially contributing to bone loss. Furthermore, long-term use of intranasal and systemic corticosteroids for managing nasal polyposis can lead to secondary osteoporosis [29].

Lastly, patients with osteoporosis and nasal polyposis often have low vitamin D levels, which plays a crucial role in bone health and immune function. Vitamin D deficiency is associated with increased inflammation and impaired immune response, linking these two conditions [30].

The main explanation for this observation lies in the limitations of our study. The primary limitation is the lack of data on the severity of osteoporosis, including BMD Z-scores and information on osteoporotic fractures. We can hypothesize that the concomitant presence of asthma and nasal polyposis might impact the severity of osteoporosis more than its prevalence.

In any case, the presence of osteoporosis in patients with severe asthma, whether with or without nasal polyps, not only significantly impacts the individual’s health but can also lead to a substantial increase in overall healthcare management costs. In 2020, Canonica et al. published a pharmacoeconomic evaluation using real-life data from the SANI registry, providing the first estimates of additional healthcare costs associated with corticosteroid-induced adverse events in patients with severe asthma. The budget impact model highlighted the considerable economic burden of OCS-related adverse events, including osteoporosis and nasal polyposis, in severe asthmatic patients [31].

However, a recent study by Latorre et al. found that in Italy, the rate of patients with severe asthma who use OCSs undergoing investigations such as bone densitometry and otolaryngological evaluation is surprisingly low, at around 7% [32]. This study highlights the ongoing need for prompt diagnosis of asthma and related comorbidities.

It is particularly crucial given that numerous strategies have been developed to improve asthma control, reduce exacerbations, and decrease the corticosteroid load in asthma management [33]. Biologic treatments, such as monoclonal antibodies that target the pathophysiological mechanisms of asthma, can control both airway and systemic inflammation, thereby positively impacting asthma comorbidities.

Biologic therapies have significantly reduced the need for systemic corticosteroids in the management of severe asthma. By targeting immunoglobulin E (IgE), thymic stromal lymphopoietin (TSLP), and specific cytokines such as IL-5, IL-4, and IL-13, biologics offer more precise and personalized treatment in patients with severe asthma and associated comorbidities [33]. By effectively controlling asthma symptoms and reducing exacerbations, biologics decrease the reliance on OCSs [34,35,36], thereby minimizing associated adverse effects, such as osteoporosis. Furthermore, growing evidence suggests that biologics may also enable a reduction in ICS doses in the treatment of asthma [37].

This innovation in asthma treatment improves patient outcomes and quality of life and reduces the long-term healthcare costs associated with chronic OCS use [38].

## 5. Conclusions

Osteoporosis is significantly associated with severe asthma, largely due to the chronic use of corticosteroids and the persistent inflammatory state of asthma. Our study adds novel real-life evidence by showing that osteoporosis is not only highly prevalent but also associated with poorer asthma control, greater exacerbation burden, and reduced lung function. These findings underscore the importance of recognizing osteoporosis risk at earlier stages of disease progression. Biologic treatments have emerged as a promising strategy to reduce dependence on OCSs in severe asthma management, helping to mitigate adverse effects such as osteoporosis, and improving patient outcomes and quality of life while potentially decreasing long-term healthcare costs.

Future research should address these aspects and elucidate the impact of comorbid inflammatory diseases, such as nasal polyposis, on osteoporosis in patients with asthma. The significant healthcare burden associated with corticosteroid-induced adverse events underscores the urgent need for effective and safer asthma management strategies, including the earlier intervention with biological therapies, guided by real-life risk factors for OCS-related diseases, such as osteoporosis, including disease duration, age, sex, cumulative steroid exposure, and asthma control.

## Figures and Tables

**Table 1:** Distribution of inhaled and biologic therapies between patients with and without osteoporosis.

	Patients with Osteoporosis	Patients Without Osteoporosis	*p*-Value
**Inhaled therapy, *n* (%)**	**282 (15.5)**	**1531 (84.4)**	0.539
ICSs + LAMAs	8 (2.8)	34 (2.2)	
ICSs + LABAs	107 (37.9)	629 (41.1)	
Triple therapy (ICSs + LABAs + LAMAs)	167 (59.2)	868 (56.7)	
**Biologic therapy, *n* (%)**	**227 (80.5)**	**1189 (77.7)**	0.327
Benralizumab	43 (18.9)	247 (20.8)	
Dupilumab	15 (6.6)	117 (9.8)	
Mepolizumab	114 (50.2)	406 (34.2)	
Omalizumab	55 (24.2)	416 (35.0)	
Tezepelumab	–	2 (0.2)	
Mepolizumab + Omalizumab	–	1 (0.1)	

Legend: ICSs: inhaled corticosteroids; LABAs: long-acting β2-agonist; LAMAs: long-acting muscarinic antagonist.

**Table 2:** Demographic and clinical characteristics.

	Patients with Osteoporosis N = 282	Patients Without Osteoporosis N = 1531	*p*-Value
**Sex, ***n* (%)			
Female	226 (80.1)	885 (57.8)	<0.001
Male	56 (19.9)	646 (42.2)	
**Age (years), ***n* (%)			
<50	21 (7.5)	471 (30.8)	<0.001
50–70	193 (68.4)	884 (57.7)	
>70	68 (24.1)	176 (11.5)	
mean (SD)	63.2 (9.9)	54.5 (13.4)	<0.001
**Age at asthma onset (years),** mean (SD)	35.6 (17.4)	32.4 (17.0)	0.007
**Age at diagnosis of asthma (years),** mean (SD)	38.8 (17.5)	35.4 (17.1)	0.003
**Years from asthma onset or diagnosis, ***n* (%)			
<10	52 (19.7)	361 (26.2)	0.008
10–20	67 (25.4)	399 (29.0)	
>20	145 (54.9)	618 (44.9)	
Not reported	18	153	
Median (IQR)	21 (12–39)	19 (9–30)	<0.001
**BMI (kg/m^2^),** median (IQR)	25.4 (22.7–28.4)	25.5 (22.8–29.2)	0.563
**Smoker, ***n* (%)			
No	198 (70.5)	1030 (68.2)	0.187
Yes	7 (2.5)	75 (5.0)	
Ex	76 (27.0)	406 (26.9)	
Pack years, median (IQR)	10 (5–16)	10 (4–20)	0.981
Not reported	1	20	
**Blood eosinophils (cell/μL),** median (IQR)	305 (100–653)	330 (120–620)	0.758
**Blood eosinophils (%),** median (IQR)	4.0 (1.5–8.8)	4.0 (1.5–8.2)	0.792
**Blood neutrophils (cell/μL),** median (IQR)	4000 (3100–5330)	3950 (3173–5198)	0.771
**Blood neutrophils (%),** median (IQR)	55.7 (47.9–62.4)	55.5 (49.1–62.7)	0.676
**Max blood eosinophils (cell/μL),** median (IQR)	685 (413–1038)	600 (350–1010)	0.314
**FeNO (ppb),** mean (SD)	47.0 (41.4)	43.9 (45.2)	0.441
**Type 2 inflammation**			
No	6 (2.6)	16 (1.4)	0.263
Yes	223 (97.4)	1163 (98.6)	
Not reported	53	352	
**Pre-bronchodilator FEV1 (L),** mean (SD)	1.7 (0.8)	2.3 (2.1)	<0.001
**Pre-bronchodilator FEV1 (%),** mean (SD)	71.2 (25.1)	75.6 (21.5)	0.007
**Pre-bronchodilator FVC (L),** mean (SD)	2.6 (1.0)	3.2 (1.0)	<0.001
**Pre-bronchodilator FVC (%),** mean (SD)	87.0 (24.9)	90.9 (20.3)	0.014
**Tiffeneau index pre-bronchodilator,** mean (SD)	66.0 (12.3)	67.4 (12.2)	0.132
**ACT**, mean (SD)	17.2 (5.3)	19.9 (5.3)	0.046
**ACQ**, mean (SD)	2.5 (1.6)	2.0 (1.6)	0.015
**Number of exacerbations in the previous 12 months,** median (IQR)	2 (0–3)	1 (0–3)	0.002
**Chronic use of OCSs, *n* (%)**			
No	225 (79.8)	1365 (89.2)	<0.001
Yes	57 (20.2)	166 (10.8)	

Legend: ACT: Asthma Control Test; ACQ: Asthma Control Questionnaire; BMI: body mass index; FeNO: fractional exhaled nitric oxide; FEV1: forced expiratory volume in 1 s; FVC: forced vital capacity; OCSs: oral corticosteroids; SD: standard deviation.

**Table 3:** Patients’ comorbidities.

	Patients with Osteoporosis N = 282	Patients Without Osteoporosis N = 1531	*p*-Value
**Rhinitis, *n* (%)**			
No	112 (40.1)	589 (38.6)	0.888
Yes, previously	33 (11.8)	188 (12.3)	
Yes, ongoing	134 (48.0)	748 (49.0)	
Not reported	3	6	
**Chronic rhinosinusitis without polyps, *n* (%)**			
No	202 (72.7)	1120 (74.6)	0.509
Yes, previously	35 (12.6)	154 (10.3)	
Yes, ongoing	41 (14.7)	228 (15.2)	
Not reported	4	29	
**Nasal polyposis, *n* (%)**			
No	151 (53.7)	831 (54.7)	0.898
Yes, confirmed by CT scan or nasal endoscopy	121 (43.1)	635 (41.8)	
Yes, suspected	9 (3.2)	54 (3.6)	
**Age at diagnosis of nasal polyposis (years), median (IQR)**	46 (33–55)	42 (33–50)	0.083
**Atopic dermatitis, *n* (%)**			
No	262 (93.6)	1411 (92.8)	0.545
Yes	12 (4.3)	85 (5.6)	
Previously	6 (2.1)	24 (1.6)	
Not reported	2	11	

## Data Availability

The data that support the findings of this study are available from the corresponding author upon reasonable request.

## Abbreviations

The following abbreviations are used in this manuscript:

ACO	Asthma–COPD overlap
ACT	Asthma Control Test
ACQ	Asthma Control Questionnaire
ATS	American Thoracic Society
BMI	Body mass index
BMD	Bone mineral density
CPRD	Clinical Practice Research Datalink
COPD	Chronic obstructive pulmonary disease
CRSwNP	Chronic rhinosinusitis with nasal polyps
DXA	Dual-energy X-ray absorptiometry
EGPA	Eosinophilic granulomatosis with polyangiitis
ERS	European Respiratory Society
FeNO	Fractional exhaled nitric oxide
FEV1	Forced expiratory volume in one second
FVC	Forced vital capacity
GINA	Global Initiative for Asthma
HES	Hospital Episode Statistics
ICSs	Inhaled corticosteroids
IgE	Immunoglobulin E
IL	Interleukin
IQR	Interquartile range
ISAR	International Severe Asthma Registry
ISTAT	Istituto Nazionale di Statistica
LABAs	Long-acting β2-agonists
LAMAs	Long-acting muscarinic antagonists
mAbs	Monoclonal antibodies
nOP	Non-osteoporosis
OCSs	Oral corticosteroids
OP	Osteoporosis
OSAS	Obstructive sleep apnea syndrome
SANI	Severe Asthma Network Italy
TNF-α	Tumor necrosis factor-alpha
TSLP	Thymic stromal lymphopoietin
WHO	World Health Organization

## Appendix A

**The SANI study group ^†^**
  Diego Bagnasco ^**16**^, Luisa Brussino ^**17**^, Cecilia Calabrese ^**18**^, Gianna Camiciottoli ^**19**^, Marco Caminati ^**12,20**^, Giovanna Elisiana Carpagnano ^**21,22**^, Cristiano Caruso ^**23**^, Angelo Guido Corsico ^**24**^, Maria Teresa Costantino ^**25**^, Claudia Crimi ^**26**^, Alice D’Adda **^27^**, Simona D’Alo ^**28**^, Maria D’Amato ^**29**^, Leda D’Amico ^**30**^, Corrado D’Andria ^**31**^, Fabiano Di Marco ^**32**^, Nicola Cosimo Facciolongo ^**33**^, Alessandro Farsi **^34^**, Manlio Milanese ^**35**^, Michele Mondoni ^**36**^, Eustachio Nettis ^**37**^, Vincenzo Patella **^38^**, Girolamo Pelaia ^**39**^, Luisa Ricciardi ^**40**^, Fabio Luigi Massimo Ricciardolo ^**41**^, Luca Richeldi **^42^**, Erminia Ridolo ^**43**^, Pierachille Santus ^**44**^, Nicola Scichilone ^**45**^, Giulia Scioscia ^**46**^, Giuseppe Spadaro ^**47**^, Antonio Spanevello ^**48**^, Paolo Tarsia ^**49**^, Massimo Triggiani ^**50**^, Mona-Rita Yacoub ^**51**^
  **^16^** Clinica di Malattie Respiratorie e Allergologia, Dip. Medicina Interna, Univ. degli Studi di Genova, IRCCS-AOU San Martino, Genoa, Italy
  **^17^** S.S.D.D.U. Immunologia Clinica e Allergologia, Ospedale Mauriziano, Torino, Italy
  **^18^** Department of Translational Medical Sciences, University of Campania “Luigi Vanvitelli”, Naples, Italy
  **^19^** UNIT ASMA GRAVE - Ambulatorio Asma Grave Pneumologia e Fisiopatologia Toraco-Polmonare, Azienda Ospedaliera Universitaria Careggi (FI), Florence, Italy
  **^20^** Department of Medicine, University of Verona, Verona, Italy
  **^21^** Department of Translational Biomedicine and Neuroscience “DiBraiN”, University of Bari Aldo Moro, Bari, Italy
  **^22^** Section of Respiratory Diseases, Policlinico Hospital of Bari, Italy
  **^23^** Allergologic Unit, Policlinico Agostino Gemelli, Rome, Italy
  **^24^** Division of Respiratory Diseases, IRCCS Policlinico San Matteo, Foundation and Department of Internal Medicine and Therapeutics, University of Pavia, Italy
  **^25^** Allergy and Clinical Immunology Unit, Department of Medicine, “Carlo Poma” Hospital Mantova, Italy
  **^26^** Respiratory Medicine Unit, Policlinico “G. Rodolico-San Marco” University Hospital, Catania, Italy
  **^27^** Broncopneumologia, Fondazione IRCCS Ca’ Granda Ospedale Maggiore Policlinico, Milan, Italy
  **^28^** UOC Allergologia - PO Civitanova Marche - AREA VASTA 3, Civitanova Marche, Italy
  **^29^** Department of Respiratory Medicine, University “Federico II” of Naples, Italy
  **^30^** UOC Pneumologia Arnas Garibaldi, Catania, Italy
  **^31^** Internal and Respiratory Medicine, SS Annunziata Hospital, Taranto, Italy
  **^32^** Pulmonary Medicine Unit, ASST Papa Giovanni XXIII Hospital, Bergamo, Italy
  **^33^** UOC di Pneumologia, Arcispedale S. Maria Nuova Azienda USL di Reggio Emilia, Italy
  **^34^** SOSD Allergology and Clinical Immunology, Ospedale S. Stefano, USL Toscana Centro, Prato, Italy
  **^35^** Pulmonology Unit, Santa Corona Hospital, Pietra Ligure (Savona), Italy
  **^36^** Respiratory Unit, ASST Santi Paolo e Carlo, San Paolo Hospital, Department of Health Sciences, University of Milan, Milan, Italy
  **^37^** Department of Emergency and Organ Transplantation, School and Chair of Allergology and Clinical Immunology, University of Bari - Aldo Moro, Bari, Italy
  **^38^** Division of Allergy and Clinical Immunology, Department of Medicine ASL Salerno, Santa Maria Della Speranza Hospital, Salerno, Italy
  **^39^** U.O. Malattie dell’Apparato Respiratorio, A.O.U. Mater Domini, Catanzaro, Italy
  **^40^** Allergologia e Immunologia Clinica, AOU Policlinico G. Martino - Università di Messina, Messina Italy
  **^41^** Department of Clinical and Biological Sciences, University of Turin, San Luigi Gonzaga University Hospital, Orbassano, Turin, Italy
  **^42^** Fondazione Policlinico Universitario A. Gemelli, IRCCS Catholic University of Rome, Italy
  **^43^** Department of Medicine and Surgery, University of Parma, Parma, Italy
  **^44^** Department of Clinical and Biomedical Sciences, University of Milan, Respiratory Diseases, Sacco University Hospital, ASST Fatebenefratelli-Sacco, Milan, Italy
  **^45^** U.O.C. Pneumologia, Azienda Ospedaliera Universitaria Policlinico P. Giaccone di Palermo, Italy
  **^46^** Malattie Apparato Respiratorio, Dipartimenti delle funzioni Mediche e Sanitarie, Azienda Ospedaliero Universitaria - Ospedali Riuniti, Foggia, Italy
  **^47^** Center for Basic and Clinical Immunology Research (CISI), University of Naples Federico II, Naples, Italy
  **^48^** Pneumologia Riabilitativa, ICS Maugeri IRCCS Tradate, Tradate (VA), Italy
  **^49^** Pneumology Unit, Grande Ospedale Metropolitano Niguarda, Milan, Italy
  **^50^** Division of Allergy and Clinical Immunology, University of Salerno, Salerno, Italy
  **^51^** Unit of Immunology, Rheumatology, Allergy and Rare Diseases, IRCCS San Raffaele Hospital, Milan, Italy
